# SARS-CoV-2 crossreactive B-cells outnumber seasonal coronavirus spike-specific clones at the end of the COVID-19 pandemic

**DOI:** 10.1038/s44298-026-00185-6

**Published:** 2026-03-19

**Authors:** Cristina Gonzalez-Lopez, Muriel Aguilar-Bretones, Julian Reinders, Jingshu Zhang, Petra van den Doel, Batuhan Bekki, Eric C. van Gorp, P. Hugo M. van der Kuy, Bart L. Haagmans, Corine H. GeurtsVanKessel, Marion P. G. Koopmans, Rory D. de Vries, Marit J. van Gils, Gijsbert P. van Nierop

**Affiliations:** 1 Department of Viroscience, Erasmus University Medical Center, Rotterdam, The Netherlands; 2 Department of Hospital Pharmacy, Erasmus University Medical Center, Rotterdam, The Netherlands; 3 Department of Medical Microbiology and Infection Prevention, Amsterdam University Medical Center, Amsterdam, The Netherlands

**Keywords:** Diseases, Immunology, Microbiology

## Abstract

How B-cell responses towards seasonal human coronaviruses (sHCoVs) impacted those towards SARS-CoV-2 has been widely studied, yet potential reverse effects are ill-defined. We compared sHCoV immune responses between cross-sectional pre-pandemic and end-pandemic cohorts of immunocompetent adults. We assessed Spike (S) reactive IgG and IgA serum and B-cell responses towards sHCoVs and dominant SARS-CoV-2 variants, and evaluated their contribution to OC43 neutralization. Pre-pandemic individuals were uniformly sHCoV IgG and IgA seropositive, yet SARS-CoV-2 S-reactivity was negligible. End-pandemic donors, had predominant SARS-CoV-2 responses that in part cross-reacted with sHCoV which accounted for higher serum NL63, HKU1 and OC43 antibody levels. This effect was strongest for OC43 S2 and this cross-reactive response contributed to OC43 serum neutralization. We conclude that SARS-CoV-2-specific immune responses impacted sHCoVs responses, particularly for OC43. This could have implications for immune protection and offers insights for the development of pan-coronavirus treatments and vaccines.

## Introduction

Population immunity towards severe acute respiratory syndrome coronavirus 2 (SARS-CoV-2) was induced by a mix of widespread circulation of the virus and coronavirus disease 2019 (COVID-19) vaccination campaigns. Low levels of pre-existing SARS-CoV-2 reactive serum antibodies and B-cells have been detected in naïve individuals, likely induced by prior infections with seasonal human coronaviruses (sHCoVs)^1,2^.

Coronaviruses^2–4^ are classified as alpha-coronaviruses (alpha-CoV), which include sHCoVs 229E and NL63, and beta-coronaviruses (beta-CoV), including sHCoVs OC43 and HKU1, all of which generally cause common cold symptoms and are repeatedly encountered from childhood onwards^2–4^. However, the beta-CoV also includes the more recently emerged and more pathogenic Middle East respiratory syndrome Coronavirus (MERS-CoV), SARS-CoV, and SARS-CoV-2. Alpha- and beta-CoVs share a variable degree of sequence and structural homology with SARS-CoV-2, which is highest with the beta-CoV^5^. Coronaviruses have a Spike glycoprotein (S) that governs attachment to and fusion with the target cell. S protrudes from the viral particle in trimeric form and consists of the membrane-proximal domain S2, containing the fusion apparatus, and the outer domain S1, containing the receptor binding domain (RBD). Homology between the S proteins of sHCoVs, SARS-CoV-2, and its variants is higher in the S2 domain than in S1, where most variability is observed in the RBD^6^.

The structural homology between SARS-CoV-2 and other coronaviruses translates into antigenic relatedness and leads to cross-reactive antibodies that predominantly target epitopes in S2, which may or may not be neutralizing^7^. Conversely, antibodies targeting S1 and RBD, especially those binding the receptor binding motif (RBM), are generally more type-specific and tend to have high neutralization potential^8^. However, several epitopes outside the RBM, located on subdominant RBD regions, are known to be conserved among different beta-CoV and may elicit cross-reactive responses^9,10^. The differential homology across S1 and S2 domains affects the balance between neutralizing and other antibodies upon heterologous viral exposures^11^. Consequently, pre-existing sHCoVs immune responses impacted the development of SARS-CoV-2 immunity upon SARS-CoV-2 infection or COVID-19 vaccination. Notably, upon primary SARS-CoV-2 infection at the beginning of the pandemic, when the general population was immunologically naïve, the magnitude of boosted OC43 S2-specific IgG responses in certain individuals positively correlated with COVID-19 severity, and these cross-reactive antibodies neutralized neither SARS-CoV-2 nor OC43^12,13^. This observation raised concerns on immunopathological interactions between SARS-CoV-2 and OC43 and supported a role for original antigenic sin (OAS) in severe COVID-19^11,14^. OAS refers to a negative attribute of immune memory whereby the immune system preferentially recalls antibodies against a previously encountered virus that is antigenically related, rather than generating a more effective virus-specific response^11^. In contrast, recent infection with OC43 has been correlated with reduced SARS-CoV-2 seroconversion rates, suggestive of cross-protection, although this was not related to OC43 S-specific IgG responses^15^. The role of OC43 in enhancing or protecting against COVID-19 remains unclear in the increasingly immune population over the course of the pandemic.

In addition, little is known about the impact of the immune interactions between sHCoVs and SARS-CoV-2 in COVID-19 convalescent individuals, as well as their impact on protection against sHCoVs, and if this changed over the course of the pandemic^16–19^. A deeper understanding of cross-reactive immune responses between sHCoVs and SARS-CoV-2 can provide insights into the antigenic relations between coronaviruses as well as host responses. This is crucial for informed development of targeted vaccines and antibody-based immunotherapies for coronaviruses, including SARS-CoV-2.

Here, we investigated the magnitude, cross-reactivity over different domains, and function of sHCoVs and SARS-CoV-2 directed antibodies in samples obtained from blood donors and healthcare workers (HCW) before and at the end of the pandemic. These were cross-sectionally sampled between 2018 and 2019 (pre-pandemic) or in February and March 2023 (end-pandemic). We explored differences in the serum IgG and IgA titers, clonal antibody cross-reactivity patterns between sHCoV and relevant dominant SARS-CoV-2 variants, and their specificity towards the S1 and S2 domains. Additionally, we determined virus neutralization titers of serum and clonal antibodies against OC43 as a measure of antibody functionality and immune protection.

## Results

### SHCoVs share structural and sequence homology with SARS-CoV-2, mostly on inner domains of S2

SHCoVs share a variable degree of sequence conservation with SARS-CoV-2, supporting potential cross-reactive T-cell and antibody responses targeting linear epitopes^5^. However, identifying potential secondary and tertiary antibody epitopes in the pre-fusion conformation of S requires combined structural and sequence analysis.

To identify amino acid residues (aa) that share structural and sequence identity, we superimposed crystal structures of sHCoVs and SARS-CoV-2 for each S1, S2, and monomer subunit in the S-trimer. The frequency of aa that could be aligned for the best fitting chains ranged between 64% and 71% for S1 and between 60% and 83% for S2 for the four sHCoV (data not shown). Next, we mapped the distance between the best pairing residues and subsequently annotated aa that shared identity on the 3D models of SARS-CoV-2 S1, S2, and S-trimer.

**Fig. 1:**
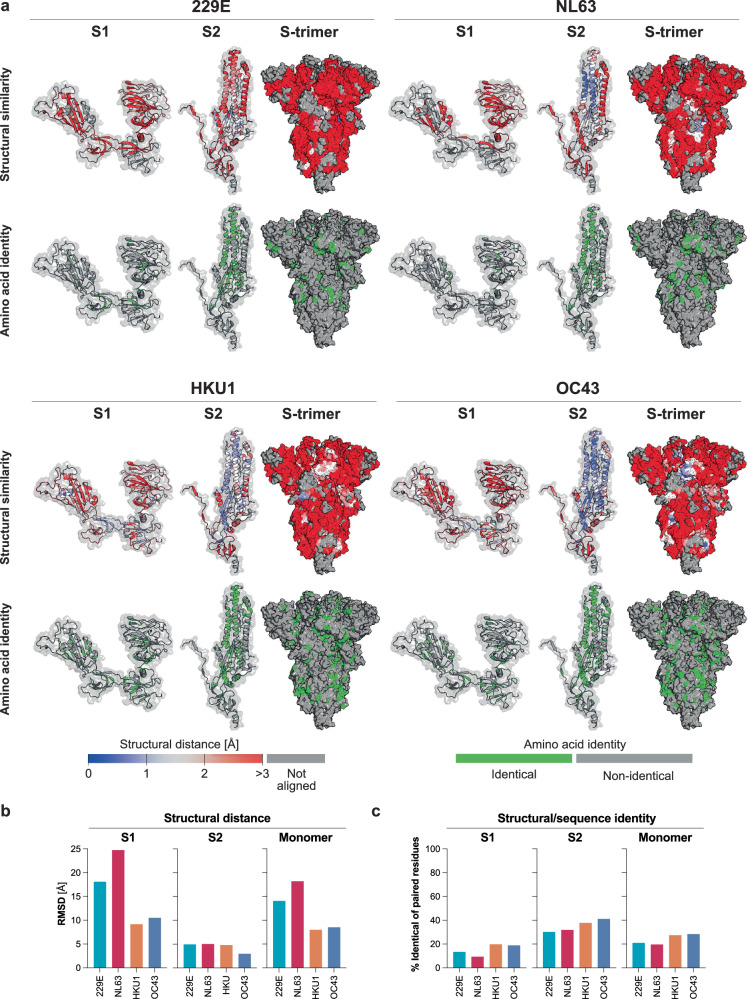
Structural and sequence comparison of SARS-CoV-2 with seasonal human coronavirus spikes. **a** Top panels: The structural distance between each overlayed amino acid in SARS-CoV-2 and its closest structural homologue in sHCoVs is mapped on a 3D model of SARS-CoV-2. The root mean square deviation (RMSD) in Ångstrom (Å), between the alpha-carbon of each amino acid (aa) of ancestral SARS-CoV-2 and seasonal human coronaviruses (sHCoVs) is color-coded from blue (overlapping; 0 Å) to red (≥3 Å). Residues that did not structurally align are depicted in grey. Bottom panels: Sequence conservation between aligned residues of SARS-CoV-2 and sHCoVs is highlighted in green. The S1 (left) and S2 domains (middle) are shown as color-coded ribbon models overlayed on a surface rendered model in grey. S-trimers (right) are shown in surface rendering to visualize exposed residues. **b** The minimum RMSD is plotted for the S1 and S2 domains and the monomer of the S-trimer that aligned best with SARS-CoV-2. **c** The frequency of identical aa out of total aa that structurally aligned best between the S1, S2, and monomer subunits of the S-trimers of SARS-CoV-2 and sHCoVs is shown.

Overall, structures in S2 were more closely aligned compared to S1. Moreover, whereas conserved/aligned aa in S1 were scattered, S2 showed larger stretches of structural conservation. Surface rendering of the S-trimer showed that the conserved aa were distributed across the antigen, yet beta-CoV showed larger patches of identically aligned aa, especially in S2. (Fig. 1a)

To quantify these measures of antigenic resemblance, we calculated the minimal root mean square deviation (RMSD) between the alpha-carbon of each paired aa for the best fitting monomers, the S1 and S2 subunit separately. The RMSD, as an inverse measure of structural homology, was higher for S1 compared to S2 for all sHCoV and higher for alpha-CoV compared to beta-CoV (Fig. 1b)

Subsequently, we quantified aligned aa that shared sequence identity between sHCoVs and SARS-CoV-2. Analogous to structural conservation, shared identity was highest for S2 compared to S1, and higher for beta-CoV compared to alpha-CoV. The highest antigenic resemblance was observed for OC43 and SARS-CoV-2 (Fig. 1c).

### Clinical characteristics of the cohorts

To experimentally determine how a potential cross-reactive immune response would develop, two cohorts were evaluated for immune recognition of sHCoVs and SARS-CoV-2 in this observational study. We compared pre-pandemic samples that were retrieved between 2018 and 2019 (*n* = 17 serum/plasma of which *n* = 8 had paired peripheral blood mononuclear cells [PBMC] samples), with end-pandemic samples that were obtained between February and March 2023 (*n* = 25 serum/plasma of which *n* = 8 had paired PBMC samples). No significant differences were found in sex, nor in age of the individuals with available information from the different groups (Supplementary Table 1).

### Serum antibody titers against NL63 S1 and OC43 and HKU1 S2 were higher in end-pandemic donors

**Fig. 2:**
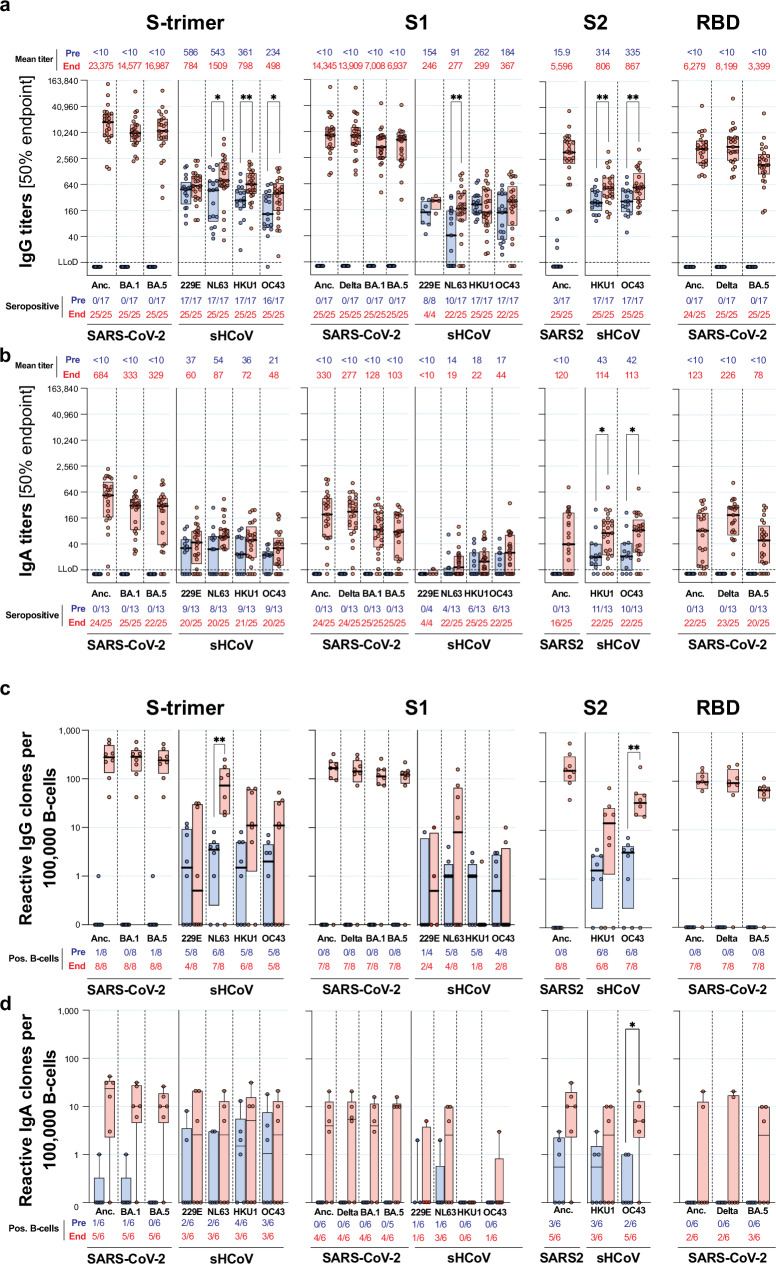
Serum IgG and IgA antibody titers and B-cell frequencies to seasonal human coronaviruses and SARS-CoV-2. **a** Serum IgG and **b** IgA 50% endpoint antibody titers towards prefusion stabilized spike (S-trimer), the S1 and S2 subdomains of spike and the receptor binding domain (RBD) of the ancestral SARS-CoV-2 strain (Anc.) and delta, omicron BA.1 and BA.5 variants and the seasonal human coronaviruses (sHCoVs) 229E, NL63, HKU1 and OC43 were determined using protein microarray from healthy individuals sampled before (blue) or at the end of the pandemic (red). Mean titers are indicated on top and the number of seroconverted individuals is indicated below the graph. The lower limit of detection (LLoD) is indicated by a dotted line. **c** The frequency of reactive IgG and **d** IgA B-cells was determined by B-cell profiling for 8 donors sampled pre- (blue) and 8 donors sampled end-pandemic (red). Center line, median; boxes, upper and lower quartiles; significance of titer differences and B-cell frequencies for sHCoVs were calculated using a Mann-Whitney test and shown as *p < 0.05 or **p < 0.01.

Serum IgG and IgA binding titers against seasonal sHCoVs and SARS-CoV-2 in pre- and end-pandemic donors were compared. Most pre-pandemic donors showed IgG titers against all four sHCoVs S-trimer and S1, as well as S2 domains of HKU1 and OC43. Interestingly, three out of 17 pre-pandemic donors (18%) had low, but detectable IgG titers against SARS-CoV-2 S2 (Fig. 2a). End-pandemic donors were all seropositive for the four sHCoVs, and SARS-CoV-2 S-specific IgG titers were dominant over sHCoV across all S domains. Titers against sHCoVs S-trimer were significatively higher in end-pandemic donors for NL63, HKU1, and OC43. For NL63, this was matched with increased titers against the S1, and for HKU1 and OC43 with increased S2 titers (Fig. 2a). A similar trend was observed for IgA, were SARS-CoV-2 titers dominated in end-pandemic donors, albeit at lower magnitude than the observed for IgG. Moreover, end-pandemic donors showed significantly higher HKU1- and OC43-S2 titers (Fig. 2b).

### Frequencies of NL63 S-trimer and OC43 S2-reactive B-cells were higher in end-pandemic donors

The frequency of B-cell clones that expressed IgG or IgA upon activation (indicative of a memory response) specific for the different coronavirus antigens was determined through B-cell profiling. The certainty of having a single reactive clone per well (clonal accuracy) per donor was high (average 91%, range 74–99%). Clonal accuracy was higher in pre-pandemic donors (97% ±2%; average ±SD) compared to end-pandemic donors (86% ±8%) due to the high frequency of S-trimer-specific clones in the latter group. Due to the lower frequency of detected S1, S2, and RBD-reactive clones, clonal accuracy for these antigens was above 95% for all donors.

In line with serological observations, in pre-pandemic donors, we mostly detected sHCoV-reactive B-cells. However, rare SARS-CoV-2 ancestral and variant-specific IgG B-cells towards S-trimer were also detected in two donors. Although all donors were seropositive for sHCoV, circulating sHCoV S trimer, S1- and S2-reactive B-cells were not detected in part of them (Fig. 2c). Nevertheless, the magnitude of the overall frequencies of antigen-specific circulating B-cells was in line with serum antibody titers (Fig. 2a/b). In correspondence with increased titers, all end-pandemic donors consistently had high frequencies of SARS-CoV-2-reactive B-cells. Moreover, the frequencies of NL63 S-trimer IgG and OC43 S2-specific IgG and IgA B-cells were higher compared to pre-pandemic donors (Fig. 2c/d). However, the frequencies in NL63 S1-specific IgG B-cells were not significantly different (Fig. 2c).

### Cross-reactive IgG to SARS-CoV-2 and sHCoV were only detected in end-pandemic donors

**Fig. 3:**
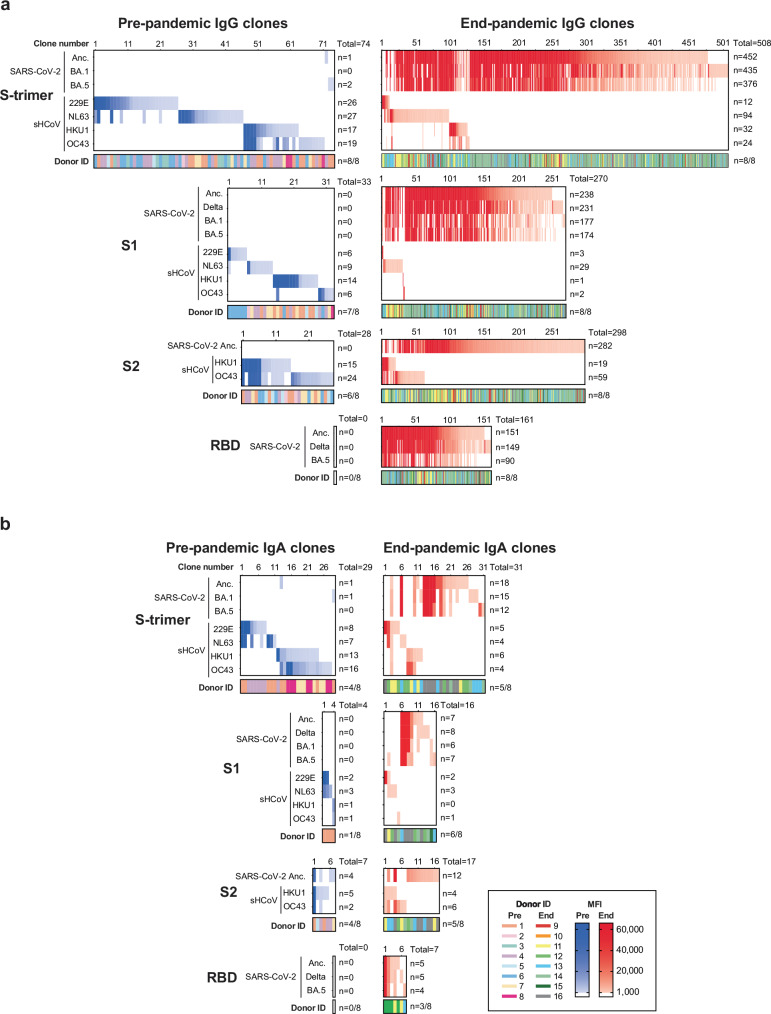
Cross-reactivity of individual IgG and IgA B-cell clones towards SARS-CoV-2 and seasonal human coronaviruses. **a** The clonal IgG cross-reactivity patterns of CD19^+^ B-cells cultured at limiting concentration were determined using B-cell profiling. The mean fluorescent intensity (MFI; range: 1000–65,350) of each clone for each antigen is indicated for pre-pandemic (blue shades) and end-pandemic individuals (red shades). The clone number, total number of reactive clones per antigen, as well as the color-coded donor ID and number of donors with reactive clones, are indicated. **b** Clonal cross-reactivity patterns of IgA clones are plotted similarly.

To study the nature of the higher sHCoV-specific antibodies and B-cell frequencies in end-pandemic donors, we analyzed the cross-reactivity patterns of reactive IgG and IgA clones. As potential tertiary epitopes are not available in monomeric antigens (S1, S2 and RBD) and the inside of S-trimer may contain cryptotopes, we stratified cross-reactivity analysis per set of S trimer, S1, S2 and RBD antigens (Fig. 3). In pre-pandemic donors, 74 IgG (Fig. 3a) and 29 IgA (Fig. 3b) reactive clones to the S trimer of the different coronaviruses were detected, of which only 3 IgG and 2 IgA clones were reactive to SARS-CoV-2.

In end-pandemic donors, 508 IgG and 31 IgA S-trimer reactive clones were detected, of which the majority were reactive and specific to SARS-CoV-2. Strong cross-reactivity between ancestral SARS-CoV-2, delta, and the omicron BA.1 and BA.5 variants was detected across S trimer, S1, and RBD. The level of cross-reactivity between alpha and beta-sHCoVs was limited, yet some cross-reactive IgG and IgA clones were detected between these two genera (Fig. 3). Strikingly, a substantial proportion of SARS-CoV-2-reactive IgG and IgA clones were cross-reactive with sHCoV. SARS-CoV-2 S1-reactive, as well as S2-reactive clones, showed cross-reactivity towards their NL63 or OC43/HKU1 counterpart antigen, respectively (Fig. 3).

### End-pandemic donors showed a shift in immunodominance towards OC43 S2

**Fig. 4:**
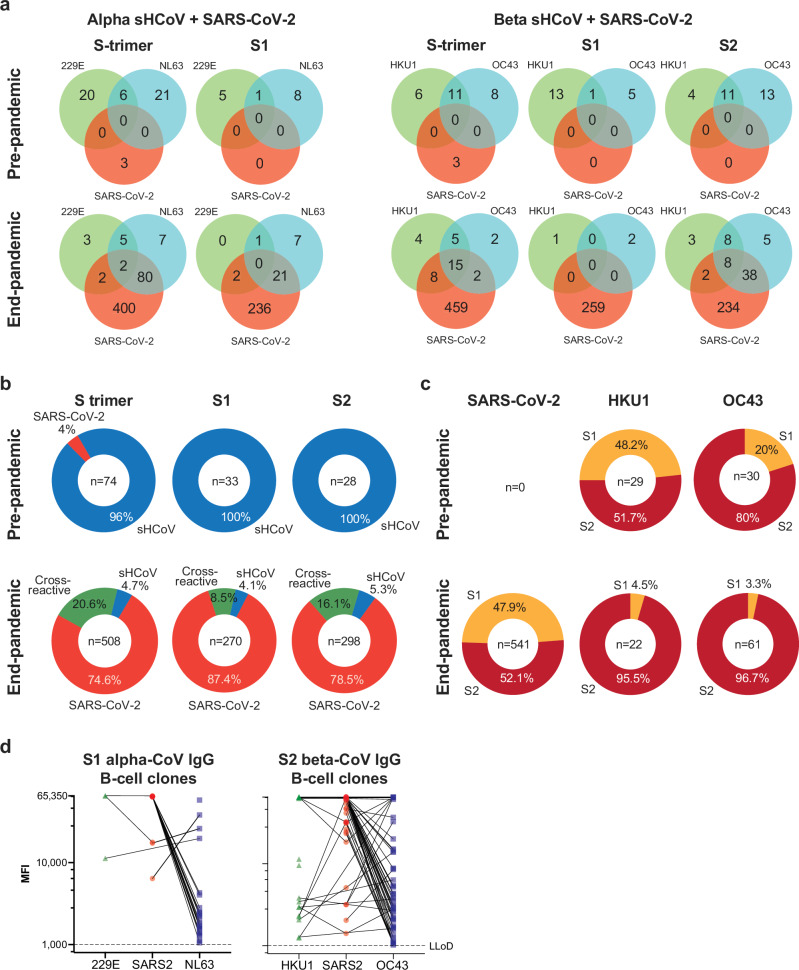
Quantification of reactive IgG B-cell clones. **a** Type specific and cross-reactive clones targeting SARS-CoV-2 and alpha-CoV or SARS-CoV-2 and beta-CoV pre- and end-pandemic are enumerated and plotted in Venn diagrams for S-trimer, S1, and S2. **b** The fraction of clones that are SARS-CoV-2-specific (red), sHCoV-specific (blue), or cross-reactive (green) from pooled pre- and pooled end-pandemic donors is plotted. **c** Proportion of beta-CoV clones, including SARS-CoV-2, that are reactive towards S1 (orange) or S2 (dark red) pre- and end-pandemic. **d** The level of reactivity of SARS-CoV-2, 229E and NL63 S1-reactive (left panel) as well as SARS-CoV-2, HKU1 and OC43 S2-reactive IgG clones (right panel) detected in end-pandemic donors is indicated as the mean fluorescent intensity (MFI; range: 1,000-65,350). Cross-reactivity of clones is indicated by a connecting line.

To further delineate the extent of these immune interactions, we quantified the number of IgG clones that react or cross-react with SARS-CoV-2 and alpha- and beta-sHCoV separately. For alpha-sHCoV, we split the analysis for S trimer and S1, and for beta-sHCoV, we split the analysis for S trimer, S1, and S2 (Fig. 4a). The higher numbers of NL63 S-trimer and S1-reactive IgG clones, and OC43 S2-reactive clones detected end-pandemic were mostly explained by the high number of clones that cross-recognized SARS-CoV-2 and their sHCoV counterpart antigen. In total, 21 cross-reactive clones targeting the S1 of SARS-CoV-2 and NL63 and 46 clones targeting the S2 of SARS-CoV-2 and OC43 were detected end-pandemic (Fig. 4a).

This increased cross-reactivity was also reflected in their relative abundance. In pre-pandemic donors, 71/74 (96%) of all S trimer-specific IgG clones targeted seasonal sHCoV. In end-pandemic donors, SARS-CoV-2-reactive IgG clones accounted for 95.2% of the total coronavirus-specific response, 20.6% of which were cross-reactive with sHCoV and 4.7% selectively reacted with sHCoV (Fig. 4b).

The high cross-reactivity impacted the relative immune dominance of S1 and S2 of beta-sHCoV. In pre-pandemic donors, the proportion of clones targeting S2 was 51.7% for HKU1 and 80% for OC43. In end-pandemic donors, SARS-CoV-2 reactive clones were evenly distributed between S1 (47.9%) and S2 (52.1%). However, because of the high proportion of cross-reactive clones targeting S2, the relative frequency of S2 targeting clones was higher for HKU1 (95.5%) and OC43 (96.7%) end-pandemic (Fig. 4c). Similar trends were observed for HKU1 and OC43 IgA clones (Supplementary Fig. 1). Altogether, this suggests a shift in target recognition towards the S2-domain of beta-sHCoV in end-pandemic donors.

### SARS-CoV-2/sHCoV cross-reactive clones preferentially reacted with SARS-CoV-2

To gain insight into the binding preference for SARS-CoV-2 or sHCoV of cross-reactive IgG clones, we compared the mean fluorescent intensity (MFI) for each clone towards each of the respective antigens. For both NL63 S1 and OC43 S2, most cross-reactive clones showed higher MFI towards the SARS-CoV-2 antigen compared to the sHCoV antigen (Fig. 4d). This suggests that out of all cross-reactive clones, most have higher binding affinity for SARS-CoV-2 than they do to the sHCoV counterpart.

### OC43 serum neutralization titers were higher in end-pandemic donors

**Fig. 5:**
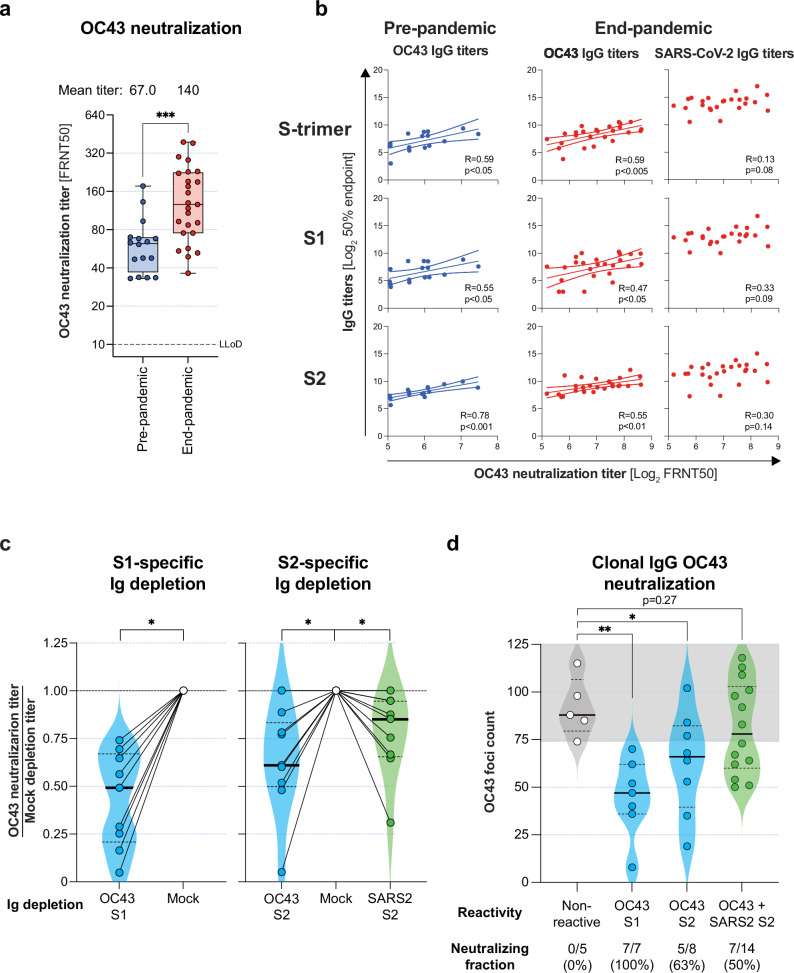
OC43 neutralization and correlations with serum IgG titers. **a** Serum OC43 neutralization potential was determined using 50% focus reduction neutralization tests (FRNT50) and compared between pre- (blue) and end-pandemic (red) donors. Statistical significance was determined by Mann–Whitney *U*-test. **b** Linear regression analysis between serum OC43 neutralization titers and OC43 IgG binding titers (pre-pandemic, blue) or OC43 and SARS-CoV-2 IgG binding titers (end-pandemic, red). The regression line and 95% confidence interval are plotted for significant correlations (*p* < 0.05). **c** The OC43 neutralization titers of sera depleted of OC43 S1-specific antibodies (left panel) (Wilcoxon paired *t*-test), and sera depleted for OC43 S2- or SARS-CoV-2 S2-specific antibodies (right panel), normalized to mock-depleted sera are shown. Statistical significance was determined between the original titers by one-way ANOVA with Geisser-Greenhouse correction. **d** IgG B-cell culture supernatants that were OC43 S1-specific, S2-specific, or SARS-CoV-2/OC43 S2-cross-reactive were tested in a OC43 focus reduction assay and compared with non-reactive controls (unpaired Student’s t-test). Significant differences were plotted as *p < 0.05, **p < 0.01 and ***p < 0.001.

To address a potential functional impact of the altered recognition of OC43 due to the increased immunodominance of S2 observed in end-pandemic donors, we assessed the serum neutralization titers using a foci reduction neutralization test (FRNT). The OC43 neutralization titers were significantly higher in the end-pandemic donors (Fig. 5a).

### SARS-CoV-2/OC43 cross-reactive S2-directed antibodies contribute to OC43 neutralization

To determine the relative contribution of OC43 and SARS-CoV-2 S-trimer, S1, and S2 IgG to OC43- neutralization, we correlated the serum IgG titers with OC43 FRNT50 titers. In both pre- and end-pandemic samples, OC43 neutralization titers correlated with OC43 S-trimer, S1, and S2 specific IgG titers. In contrast, correlations with SARS-CoV-2 IgG titers in end-pandemic donors were not significant (Fig. 5b). Because only a minor fraction of the total SARS-CoV-2 S2-directed clones cross-reacted with OC43 (46/282 clones; 16.3%, Figs. 3a and 4a), such correlations may be difficult to identify in a mixed population of antibodies.

Therefore, we subsequently assessed the relative contribution of OC43 S1 and S2-specific antibodies and SARS-CoV-2 S2 cross-reactive antibodies to OC43 neutralization by comparing sera depleted for these specific antibody fractions with mock-depleted sera on OC43 FRNT. For samples where surplus material was available, the efficacy and specificity of antigen-specific depletions were confirmed by IgG enzyme-linked immunosorbent assay (ELISA) for the homologous compared to a heterologous antigen, respectively (Supplementary Fig. 2).

All depleted sera showed reduced OC43 FRNT titers compared to mock, but the differences for OC43 S2 and SARS-CoV-2 S2 were relatively small compared to OC43 S1 (Fig. 5c). This suggested that not only OC43-specific antibodies, but also SARS-CoV-2 S2 cross-reactive antibodies contributed to the total OC43 neutralization potential of serum, albeit to a limited extent.

### OC43 S1 and S2-specific IgG clones showed OC43 neutralization potential

To investigate the OC43 neutralization potential of OC43 S1 and S2-specific, and SARS-CoV-2/OC43 S2-cross-reactive IgG clones further, we determined the OC43 neutralization potential of surplus culture supernatants using an adapted OC43 focus reduction assay. All OC43 S1 reactive clones (7/7; 100%) and 5 out of 8 (63%) OC43 S2-reactive clones showed a variable degree of OC43 foci reduction compared to non-reactive supernatants, which for each group of reactive clones was significantly differed from non-reactive clones (*p* < 0.01 for S1 and *p* < 0.05 for S2, respectively; Fig. 5d). For SARS-CoV-2/OC43 S2-cross-reactive clones, 7/14 (50%) showed a reduction in the number of detected OC43 foci compared to the negative control, suggesting that part of the SARS-CoV-2/OC43 S2-cross-reactive clones had neutralization potential. However, taken together, the collection of cross-reactive clones was not significantly different from non-OC43 reactive clones (*p* = 0.27; Fig. 5d), which may be caused by limited statistical power in this sub-analysis or heterogeneity in the neutralization potential of S2 cross-reactive clones. When analyzing OC43 S2-specific and SARS-CoV-2/OC43 S2-cross-reactive B-cells together, the reduction of OC43 foci was also not significant (*p* = 0.22, data not shown), suggesting that overall, OC43 S2-specific clones had higher neutralization potential compared to SARS-CoV-2/OC43 S2-cross-reactive IgG. Nevertheless, together with the immunodominance of cross-reactive clones, these analyses supported that part of the SARS-CoV-2/OC43 S2-cross-reactive IgG clones contributed to the higher serum OC43 neutralization titers observed in end-pandemic donors.

## Discussion

The clinical impact of the complex immune interactions between sHCoVs and SARS-CoV-2 on immune protection has been debated^12,13,15,20–23^. There are indications for OC43-induced OAS in severe COVID-19 patients after primary SARS-CoV-2 infection early in the pandemic^12,13,20,22,23^. Yet, how these immune interactions have evolved over time in the general adult population, where the SARS-CoV-2 immune status has become increasingly diverse and where only a fraction suffered from severe COVID-19, is poorly understood. In this study, we retrospectively compared the immunorecognition of sHCoVs and SARS-CoV-2 and their interplay before and at the end of the COVID-19 pandemic in cross-sectionally sampled adult blood donors who either had no or presumably had multiple exposures to SARS-CoV-2 through vaccination and/or infection, respectively.

Sequence alignment between SARS-CoV-2 and sHCoV S suggests that HKU1 is most like SARS-CoV-2^5^, implying stronger antigenic relatedness for T-cells and B-cell targeting linear epitopes. The comparative structural and sequence analysis between sHCoV and SARS-CoV-2 suggested the presence of potential shared conformational secondary or tertiary epitopes, especially between OC43 and SARS-CoV-2 S2, which may lead to the induction of cross-reactive immune responses. We confirmed these B-cell immune interactions by unbiased clonal analysis of the B-cell response. Compared to the pre-pandemic donors, we showed higher NL63 S1, HKU1 S2, and OC43 S2 specific antibody titers in end-pandemic donors, which is in line with previous findings^17–19^. In contrast to OC43, no associations with severe COVID-19 have been described for NL63 and HKU1. Conversely, no aberrancies in medically attended or hospitalized sHCoV cases were reported over the SARS-CoV-2 pandemic^24^. These observations suggest that the net impact of the COVID-19 pandemic on the severity of infection with sHCoVs was neither significantly detrimental nor beneficial at the population level.

The antigenic relatedness between NL63 S1 and SARS-CoV-2 S1 is currently not clear. Our structural and sequence analysis is in line with previous work showing distinct orientations of the C-terminal and N-terminal domains in S1, which results in limited structural and sequence alignment, despite the similar use of ACE-2 as an entry receptor^25,26^. Although serum IgG titers and cross-reactivity were increased in end-pandemic samples, the frequency of IgG B-cells remained similar. The relatively low binding capacity of cross-reactive clones to NL63 suggested that this immune interaction was not substantial.

For HKU1, the level of structural and sequence homology with SARS-CoV-2 within S2 was comparable with that of OC43^5,27,28^. Despite these similarities, the number of detected SARS-CoV-2/HKU1 cross-reactive B-cells was relatively low compared to OC43. Previously, depletion of S2-directed antibodies in convalescent sera yielded a significant reduction to the antibody binding of OC43 S, but had little effect on HKU1 S binding antibodies^18^. These discrepancies are likely related to limited affinity of cross-reactive antibodies. Taken together, this suggests a lower contribution of SARS-CoV-2 S2-directed antibodies in HKU1 S binding compared to OC43.

The detected higher OC43 S2 IgG titers and frequency of OC43 S2-reactive B-cells in end-pandemic donors is reminiscent of the observed boost of OC43 B-cells in severe COVID-19 patients^12,13^. However, while in severe COVID-19 patients, the cross-reactivity of boosted OC43 S2-reactive towards SARS-CoV-2 was limited, we observed that SARS-CoV-2/OC43 S2-cross-reactive clones preferentially targeted SARS-CoV-2. This poses questions on the heritage and maturation of cross-reactive antibodies as OC43-induced clones may acquire reactivity towards SARS-CoV-2 by affinity maturation, or SARS-CoV-2 may induce clones that are by default cross-reactive or acquire this potential by affinity training after OC43 exposure^12^. Although the preferential recognition of SARS-CoV-2 suggests that S2-cross-reactive antibodies detected here were induced by SARS-CoV-2 infection or vaccination, claims on heritage and affinity maturation would need to be substantiated with longitudinal tracing of clones using BCR sequencing to address somatic hypermutations combined with antibody affinity measurements.

Functional differences between OC43-reactive antibodies after primary SARS-CoV-2 infection and/or COVID-19 vaccination are not yet well understood. COVID-19 convalescent and vaccinated individuals are both known to have increased OC43 binding titers^19,29,30^. When comparing convalescent and vaccinated donors, the first had higher OC43 antibody-dependent cellular cytotoxicity, complement deposition, and phagocytosis compared to vaccinated-only donors^31^. In addition, vaccinated donors with a subsequent breakthrough SARS-CoV-2 infection had higher OC43 neutralizing activity in serum and saliva upon recovery^17^. Although the exposure history of end-pandemic donors in our study is undefined, the increased OC43 binding and neutralizing antibody titers described here are in line with these observations.

Differences in antibody functionality between groups may result from conformational differences in SARS-CoV-2 S. Whereas mRNA vaccination selectively elicits a response towards a proline-stabilized prefusion spike, infection may induce a response to a much wider range of S conformations that additionally expose epitopes within the fusion machinery located in S2. While certain epitopes accessible in an open or post-fusion conformation of S in S2 are targeted by non-neutralizing antibodies^32^, antibody binding epitopes in the pre-fusion stem-helix of SARS-CoV-2 S2 correlate with broad neutralization against beta-CoVs^7^^,^^33–36^ and were prevalent in convalescent vaccinated individuals but were scarce in vaccinated-only and convalescent-only individuals^7^. This highlights the functional heterogeneity of S2-directed antibodies depending on conformational target epitopes, which corresponds with the variable OC43 neutralization potential of S2-reactive clones that we observed.

OC43 S1-specific IgG showed highest OC43 neutralization potential in serum and at a clonal level. Although not significantly different, slightly higher levels of OC43 S1-specific serum IgG titers and B-cell frequencies were detected in end-pandemic individuals, which likely contributed to the increased serum OC43 neutralization titers. In addition to that, despite the relatively low OC43 binding affinity and OC43 neutralization potential of SARS-CoV-2/OC43 S2-cross-reactive antibody clones, their increased abundance supports their contribution to the increased serum OC43 neutralization observed in end-pandemic donors. This was confirmed with the slightly reduced OC43 neutralization potential of sera depleted for the respective antibody fractions.

Whether the higher neutralizing titers lead to improved immune protection against OC43 infections remains to be determined. Notably, the dominance of SARS-CoV-2/OC43 S2 cross-reactive clones over OC43 S2-specific clones, combined with preferential binding of cross-reactive clones towards SARS-CoV-2 S2 observed in end-pandemic donors, could lead to prozone effects, where an excess of lower affinity cross-reactive antibodies outcompetes more potent type-specific responses. Indeed, our clonal OC43 focus reduction analysis suggested that the S2 cross-reactive IgG had lower binding and neutralization potential compared to OC43 type-specific IgG. Yet, the large number of cross-reactive clones may compensate for their lower potency. On the other hand, increasingly potent cross-reactive clones may be selected by repeated heterologous exposures and affinity maturation that have an increasingly beneficial role by providing broad immune protection. Therefore, continued longitudinal studies on the balance between type-specific and cross-reactive responses and how cross-reactivity evolves in the context of continued exposures to sHCoVs and SARS-CoV-2 remain of interest.

Limitations of this retrospective, cross-sectional study design are the limited study size and the lack of paired pre-/end-pandemic samples, which restricts extrapolation of our results to the general population. Also, there was no clinical information on recent SARS-CoV-2 and/or sHCoV exposure of the study groups. The higher OC43 neutralization titers in end-pandemic donors could be a direct result of the induction of a SARS-CoV-2/OC43 cross-reactive response or by recent exposure to OC43. To investigate potential biases in OC43 exposure at a population level, we compared local epidemiological surveillance data^24^. Seasonal patterns of sHCoV detections were disturbed from the end of 2020 to early 2022 due to lock-down measures, yet circulation persisted at low levels. After that, both the number of detections and seasonal pattern reverted to similar levels observed pre-pandemic. Nevertheless, according to epidemiological data, end-pandemic donors were sampled between 10 and 15 weeks after the peak of reported sHCoV infections in the Netherlands, whereas pre-pandemic donors were more scattered. These observations indicate there is a potential risk of bias based on recent OC43 exposure (Supplementary Fig. 3)^24^. In contrast, SARS-CoV-2 S1- and RBD-reactive antibodies decay faster than S2-directed antibodies upon infection. An increased ratio of S1/S2-reactive antibodies is therefore indicative of recent SARS-CoV-2 exposure^37,38^. Assuming similar differential kinetics for OC43 S1 and S2, the similarities in OC43 S1-directed antibody titers and frequencies compared to pre-pandemic donors, and increased S2-directed IgG response argue against recent OC43 exposure.

In conclusion, the unparalleled number of antibody clones analyzed here for their cross-reactivity potential and function offers a detailed insight in coronavirus immune interactions. Our data shows that SARS-CoV-2 has altered immune recognition of NL63, HKU1, and OC43, due to antibody cross-reactivity. The increased IgG cross-reactivity with OC43 S2 and the higher levels of OC43 neutralization in end-pandemic donors are of particular interest. Despite the OC43-related immunopathogenic effect in severe COVID-19 donors observed early in the pandemic, our data on individuals sampled at the end of the pandemic support that SARS-CoV-2 S2-reactive B-cells and antibodies positively contribute to OC43 neutralization. Whether this translates into immune protection remains to be confirmed, but in that scenario, S2 might constitute a relevant target for the design of pan-corona vaccines.

## Methods

### Comparative structural and sequence analysis

The crystal structures of S-trimers obtained from the protein databank of 229E (protein data bank [PDB] 7cyd), NL63 (PDB 5SZS), HKU1 (PDB 8ohn), and OC43 (PDB 9blk) were superimposed on ancestral SARS-CoV-2 (PDB 9bd9) for each S1 and S2 subunit and monomer separately using PyMOL (Schrödinger, Inc.). Structural alignment was quantified by calculating the minimum RMSD between the alpha-carbons of structurally resolved amino acids for each combination of S1, S2 subunits, and monomer separately. The maximum frequency of paired amino acids that shared identity and structural position was calculated for the best aligned monomer, S1 and S2, for all sHCoVs and SARS-CoV-2. The RMSD and shared amino acids were mapped on SARS-CoV-2 S1, S2, and S-trimer.

### Study design and clinical specimens

Blood samples were either collected as surplus material from blood donations at the Sanquin Blood Bank (Rotterdam, the Netherlands), or as cross-sectional samples from the SWITCH-ON study (ClinicalTrials.gov ID: NCT05471440, submitted 17 July 2022) or the COVA biobank study (Supplementary Table 1)^39^. The study protocols were approved by the Medical Ethics Committee of the Erasmus University Medical Center, Rotterdam, The Netherlands (SWITCH-ON: MEC-2022-0462; COVA: MEC-2014-398) and were conducted in accordance with the Declaration of Helsinki. Study participants were either adult voluntary blood donors (8 donors pre-pandemic and 4 end-pandemic) or HCW part of the COVA biobank study (9 donors pre-pandemic, samples collected in November 2018) or the SWITCH-ON COVID-19 vaccination study (21 donors end-pandemic, samples collected in March 2023). Informed consent was obtained from all study subjects. Samples from the pre-pandemic group were collected between 2018 and 2019, prior to the beginning of the COVID-19 pandemic (*n* = 17 serum/plasma, of which *n* = 8 had paired PBMC samples). Samples from the end-pandemic group were collected in February or March 2023 (*n* = 25 serum/plasma, of which *n* = 8 had paired PBMC samples), at the end of the pandemic. PBMC were isolated from buffy coats, EDTA or heparin blood samples using Lymphoprep density-gradient (Stemcell Technologies), and if available, serum was isolated from serum tubes according to the manufacturer’s instructions. Serum/plasma and PBMC samples were cryopreserved in aliquots at −20 °C and −196 °C for subsequent analysis, respectively.

### Protein microarray

For multiplex serology and B-cell analysis, a custom protein microarray (PMA) was generated as previously described^12^. The PMA consisted of a panel of S-trimer, S1, S2, and selected RBD antigens from the ancestral SARS-CoV-2 strain and relevant dominant variants (Delta, Omicron BA.1 and Omicron BA.5), as well as S-trimer and the S1 from the four endemic sHCoVs (229E, NL63, HKU1, and OC43), in addition to the S2 of HKU1 and OC43. 229E S1 reactivity was not determined for all donors because this antigen did not pass quality control. All antigens were obtained commercially (SinoBiological) except for the S-trimer antigens of sHCoVs, which were generated in-house as previously described (Supplementary Table 2)^18^. The minimal antigen concentration that provided stable EC50 titers titrated for each antigen, was used. As differences between tested variants and strains were minimal per antigen type (S-trimer, S1, S2, and RBD), equimolar amounts were printed per antigen type (data not shown).

### Serology

Serum and plasma samples were used interchangeably to determine IgG and IgA binding titers against all antigens on the PMA as previously described^12^, and referred to as serum throughout for convenience. In brief, PMA slides were blocked with Blotto (ThermoFisher Scientific) for 1 h at 37 °C and washed with PBS + 0.01% Tween (Sigma Aldrich; PBST). Four-fold dilution series of plasma were prepared in Blotto, starting at 1:10 and up to 1:163,840, and loaded onto the blocked and washed PMA slides for 1 h at 37 °C. Subsequently, slides were washed 4 times with PBST and stained with Alexa647-conjugated AffiniPure Goat anti-Human IgG Fcγ specific antibody (Jackson ImmunoResearch Laboratories; 109-605-008) or Cy3-conjugated AffiniPure Goat anti-Human IgA α-chain specific antibody (Jackson ImmunoResearch Laboratories; 109-165-011) in Blotto for 1 h at 37 °C. After staining, slides were washed 4 times with PBST, once with ddH_2_O, and airdried. PMA slides were scanned in a PowerScanner (Tecan). Serum IgG and IgA titers were determined as the EC50 using a 4-parameter logistic regression with GraphPad Prism v10.

### B-cell profiling

Short-term B-cells were cultured at limiting density to ensure clonal reactivity as described previously^12,40^. Briefly, B-cells were isolated from cryopreserved PBMCs using EasySep Human CD19 Positive Selection Kit (STEMCELL Technologies) according to manufacturer’s instructions. Cultures of 100-400 B-cells per well (pre-pandemic) or 50-100 B-cells per well (end-pandemic) were seeded in 96-well U-bottom plates in AIM-V AlbuMAX medium supplemented with 10% FCS, penicillin-streptomycin-glutamate (PSG; Invitrogen, ThermoFisher Scientific), and β-mercaptoethanol (Invitrogen) (B-cell medium) (Supplementary Table 3). B-cell cultures were stimulated in a B-cell receptor-independent manner for 72 h with 50 U/mL IL-2 (Novartis), 10 ng/mL IL-10 (Peprotech), 25 ng/mL IL-21 (Peprotech), 1 μg/mL resiquimod (Invivogen), and 10 times more 40 Gy x-ray irradiated, growth arrested L-CD40L cells (provided by J. Banchereau, The Jackson Laboratory for Genomic Medicine, Farmington, Connecticut, USA) than the number of CD19^+^ B-cells. These cytokines, TLR agonists and CD40L stimulated both IgD naïve and memory IgM, IgG and IgA B-cell to proliferation and differentiation^41,42^. CD40L expression and the absence of mycoplasma were confirmed for L-CD40L cells. After 72 h, B-cells were cultured in B-cell medium supplemented with 25 ng/mL IL-21 for up to 14 days. Finally, culture supernatants were stored at −20 ˚C until the measurement of IgG and IgA reactivity using PMA. We refer to the antibodies secreted in supernatant from oligoclonal B cell cultures as ‘clones’ in this manuscript.

Culture supernatants were mixed 1:1 with Blotto and reactivity was analyzed using PMA. Bound IgG and IgA were stained fluorescently, analogous to serology. The mean MFI (range 0–65,350) of Alexa647-conjugated AffiniPure Goat anti-Human IgG Fcγ specific antibody (Jackson ImmunoResearch Laboratories; 109-605-008) and Cy3-conjugated AffiniPure Goat anti-Human IgA α-chain specific (Jackson ImmunoResearch Laboratories; 109-165-011) was measured using a Powerscanner. A cut-off of 1000 MFI was set for all antigens, except for NL63 S1. This antigen gave higher background which was corrected by setting a batch-dependent cut-off (3000 for batch 1 and 6000 for batch 2, respectively) based on the MFI of non-reactive cultures (Supplementary Fig. 4).

The frequency of reactive B-cells was calculated by counting the number of reactive wells divided by the total number of wells screened, multiplied by the number of cells per well. The frequency of reactive wells was used to calculate the probability of monoclonality (clonal accuracy) based on the Poisson distribution^43^.

### Serum S1 and S2-specific antibody depletion

His-tagged OC43 S1, S2, and SARS-CoV-2 S2 antigens (Supplementary Table 2) were coupled to HisPur Ni-NTA Magnetic beads (Thermo Fisher) according to manufacturer’s instructions. For S1, 1 µg antigen was coupled to 10 µl bead suspension, and for S2, 2 µg antigen was coupled to 14 µl bead suspension, prepared fresh for each depletion. 1% BSA blocked beads were used as a mock depleted control. Sera were heat inactivated at 56 °C for 30 min and diluted 1:10 in Opti-MEM (Thermo Fisher) with PSG and incubated with coated magnetic beads for 1 h under continuous mixing on a Rollerbank (IKA) at room temperature, after which the beads were removed using a BD IMag magnet (BD Biosciences). The depletion procedure was repeated twice. Efficiency and specificity of depletions were validated by performing IgG titrations of the homologous and a heterologous antigen, respectively. IgG levels were quantified through ELISA as described previously^12,44^.

### OC43 propagation and neutralization assay

The OC43 strain VR-1558 (ATCC, CCL-244) was propagated in MRC-5 cells (ATCC, CCL-171). Briefly, cells were infected at a multiplicity of infection (MOI) of 0.01. Progeny viruses were harvested 6 days post-infection and concentrated at 20,000 × *g* for 1.5 h at 4 °C hrough a 10% sucrose cushion.

Focus Reduction Neutralization Test (FRNT) assays were performed in A549 cells (ATCC, CCL-185). Sera samples were heat-inactivated for 30 min at 56 °C. Undepleted or S1/S2-specific antibody depleted sera were then 3-fold serially diluted in 60 μL of Opti-MEM I (1×) + 1% penicillin-streptomycin (Gibco), starting at 1:20 in a 96-well V-bottom plate. Alternatively, B-cell culture supernatants were mixed 1:1 with Opti-MEM. Approximately 500 or 100 OC43 infectious viral particles in a volume of 60 μL were subsequently added to the diluted serum or B-cell culture supernatant samples and incubated for 1 h at 37 °C, respectively. The virus-serum or virus-supernatant mixtures (100 μL) were then transferred to confluent monolayers of A549 cells and incubated overnight at 37 °C. Cells were fixed in 4% formalin, permeabilized with 70% ethanol, and blocked in 0.6% bovine serum albumin (BSA, Sigma) in PBS for 30 min. Cells were then incubated with rabbit anti-OC43 nucleocapsid antibody (Sino Biological, 40643-762, 1: 2000 in 0.1% BSA), followed by incubation with Alexa Fluor 488-conjugated goat anti-rabbit IgG (Invitrogen, A32731, 1: 2000 in 0.1% BSA). Imaging was performed using the Amersham Typhoon Biomolecular Imager. Infected cells were quantified with ImageQuantTL 8.2.0.0 (GE Healthcare). The 50% foci reduction serum titers (FRNT50) were calculated using a 4-parameter logistic regression with Graphpad Prism v10. For B-cell culture supernatants, the number of foci were compared directly. The lowest number of foci observed among the OC43 non-reactive supernatants was used as a cut-off distinguish neutralizing from a non-neutralizing supernatant. Pooled intravenous immunoglobulin preparations from healthy blood donors were included in all assays as a positive control (data not shown).

### Statistics

Normality of data distribution was tested using Kolmogorov–Smirnov test to decide for parametric vs non-parametric test. Mann–Whitney tests were used to compare serology titers and reactive B-cell frequencies targeting sHCoV antigens and to compare OC43 neutralization titers between the pre- and end-pandemic groups. As this was not a primary readout of our analyses, the differences in SARS-CoV-2 IgG and IgA titers and the respective B-cell frequencies were not statistically analyzed. Wilcoxon–test was used to compare OC43 S1 versus mock-depleted sera. One-way ANOVA with Geisser-Greenhouse correction was used to compare OC43 S2 and SARS-CoV-2 S2 versus mock-depleted sera. For supernatants, differences in OC43 neutralization were compared to the negative control group using Student’s t-test. For correlation analyses, the Pearson coefficient was calculated. All statistical analyses were carried out using GraphPad Prism v10 (GraphPad Prism Software).

## Supplementary information

Supplemental Table and Figure

## Data Availability

Upon a reasonable request, the corresponding author can supply the data in this study.
