# Supplementary material for: SARS-CoV-2 crossreactive B-cells outnumber seasonal coronavirus spike-specific clones at the end of the COVID-19 pandemic
Source: Npj Viruses. 2026 Mar 19;4:19. doi: 10.1038/s44298-026-00185-6 (PMC13003012; doi:10.1038/s44298-026-00185-6)
Supplement: Supplementary file 1 — Supplemental Table and Figure [file 44298_2026_185_MOESM1_ESM.pdf]

Supplementary material to:

# SARS-CoV-2 crossreactive B-cells outnumber seasonal coronavirus spike-specific clones at the end of the COVID-19 pandemic

Cristina Gonzalez Lopez[1], Muriel Aguilar-Bretones[1], Julian Reinders[1], Jingshu Zhang[1], Batuhan Bekki[1], Petra van den Doel[1], Eric C. van Gorp[1], P. Hugo M. van der Kuy[2], Bart L. Haagmans[1], Corine H. GeurtsVanKessel[1], Marion P.G. Koopmans[1], Rory D. de Vries[1], Marit J. van Gils[3] and Gijsbert P. van Nierop[1]*

[1]Department of Viroscience, Erasmus University Medical Center, Rotterdam, The Netherlands
[2]Department of Hospital Pharmacy, Erasmus University Medical Center, Rotterdam, the Netherlands
[3]Department of Medical Microbiology and Infection Prevention, Amsterdam University Medical Center, The Netherlands
*Correspondence to: g.p.vannierop@erasmusmc.nl, Tel: +31 10 7044068

**Supplementary table 1.** Clinical characteristics of the cohorts.

| Cohort Characteristics | | | | | |
|---|---|---|---|---|---|
| **Pre-pandemic** | | | | | |
| **Study** | **Sex** | | **Age (years)** | **Serum n = 17** | **PBMC n = 8** |
| | **Female** | **Male** | | | |
| COVA Biobank n = 9 | 2 | 7 | 45 ± 5 | 9 | 0 |
| Blood bank n = 8 | N.D. | N.D. | N.D. | 8 | 8 |
| **End-pandemic** | | | | | |
| **Study** | **Sex** | | **Age (years)** | **Serum n = 25** | **PBMC n = 8** |
| | **Female** | **Male** | | | |
| SWITCH-ON n = 21 | 10 | 10 | 44 ± 6 | 21 | 4 |
| Blood bank n = 4 | N.D. | N.D. | N.D. | 4 | 4 |

Age and sex data from n=17 individuals obtained between 2018 and 2019 (pre-pandemic) and n=25 individuals obtained between February and March 2023 (end-pandemic) are shown. No differences were observed regarding age and sex between groups. Data of blood bank donors were unavailable due to data protection policies; therefore these were not determined (N.D.).

**Supplementary Table 2.** Antigens on protein microarray.

| Virus (variant) | Antigen | Cat.No/UniProt ID | Supplier |
|---|---|---|---|
| SARS-CoV-2 (Ancestral) | S-trimer | 40589-V08H8 | SinoBiological |
| | S1 | 40591-V08H3 | SinoBiological |
| | S2 | 40590-V08B | SinoBiological |
| | RBD | 40592-V08H | SinoBiological |
| SARS-CoV-2 (Delta) | S1 | 40591-V08H23 | SinoBiological |
| | RBD | 40592-V08H90 | SinoBiological |
| SARS-CoV-2 (Omicrons BA.1) | S-trimer | 40589-V08H26 | SinoBiological |
| | S1 | 40591-V08H41 | SinoBiological |
| SARS-CoV-2 (Omicrons BA.5) | S-trimer | 40589-V08H32 | SinoBiological |
| | S1 | 40591-V08H46 | SinoBiological |
| | RBD | 40592-V08H130 | SinoBiological |
| HCoV-229E | S-trimer | NP_073551 | In-house[1] |
| | S1 | 40601-V08H | SinoBiological |
| HCoV-NL63 | S-trimer | AKT07952 | In-house[1] |
| | S1 | 40600-V08H | SinoBiological |
| HCoV-HKU1 | S-trimer | Q0ZME7 | In-house[1] |
| | S1 | 40021-V08H | SinoBiological |
| | S2 | 40021-V08B | SinoBiological |
| HCoV-OC43 | S-trimer | Q696P8 | In-house[1] |
| | S1 | 40607-V08H1 | SinoBiological |
| | S2 | 40607-V08B1 | SinoBiological |

All antigens were produced in HEK293 cells with a C-terminal His-tag, except S2 antigens. These were produced in Baculovirus-insect cells. [1]In-house proteins were produced as described in (Grobben et al., 2021a)

**Supplementary table 3.** Positive B-cells per donor.

| Donor | Positive wells (IgG) | Positive wells (IgA) | Number of wells | Total number of cells |
|---|---|---|---|---|
| **Pre-pandemic** | | | | |
| Donor 1 | 40 | 12 | 640 | 201.600 |
| Donor 2 | 3 | 0 | 192 | 76.800 |
| Donor 3 | 8 | 0 | 192 | 76.800 |
| Donor 4 | 20 | 10 | 192 | 76.800 |
| Donor 5 | 7 | 1 | 512 | 163.200 |
| Donor 6 | 21 | 0 | 416 | 110.400 |
| Donor 7 | 13 | 8 | 416 | 121.600 |
| Donor 8 | 3 | 7 | 96 | 38.400 |
| **End-pandemic** | | | | |
| Donor 1 | 23 | 0 | 192 | 9.600 |
| Donor 2 | 4 | 0 | 192 | 9.600 |
| Donor 3 | 75 | 8 | 192 | 9.600 |
| Donor 4 | 56 | 10 | 192 | 9.600 |
| Donor 5 | 90 | 13 | 192 | 19.200 |
| Donor 6 | 222 | 8 | 768 | 62.400 |
| Donor 7 | 12 | 1 | 96 | 9.600 |
| Donor 8 | 55 | 10 | 192 | 19.200 |

Eight pre-pandemic and eight end-pandemic donor samples were analyzed through B-cell profiling. Number of positive wells for IgG and IgA, number of analyzed wells, as well as total number of analyzed B-cells are shown.

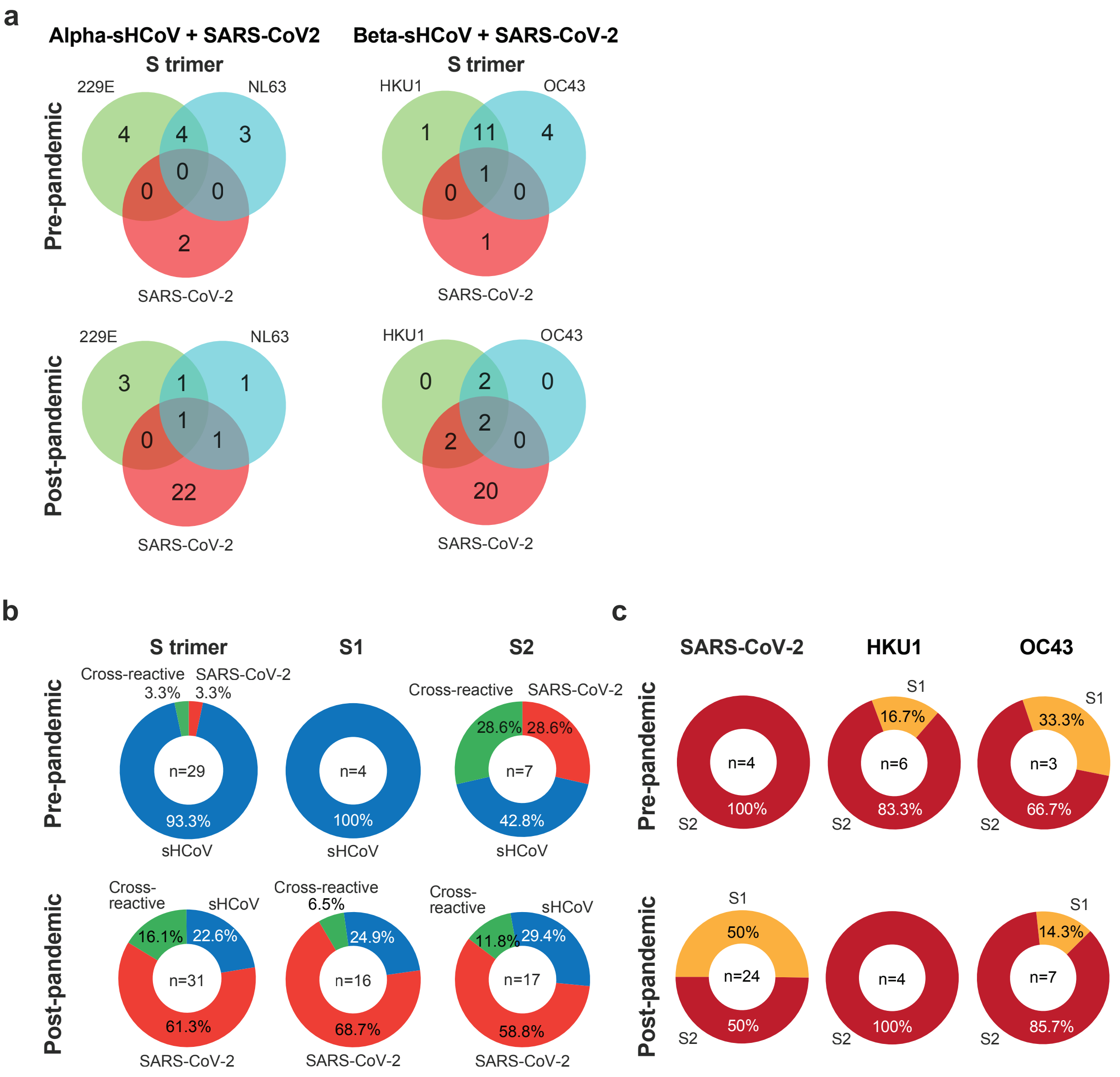


**Supplementary Figure 1. Quantification of reactive IgA B-cell clones. a**) Type-specific and cross-reactive clones targeting alpha-sHCoV and SARS-CoV-2 or beta-sHCoV and SARS-CoV-2 pre- and end-pandemic are enumerated and plotted in Venn diagrams. **b**) The fraction of clones that are SARS-CoV-2-specific (red), sHCoV-specific (blue) or cross-reactive (green) from pooled pre- (top panels) and pooled end-pandemic (bottom panels) donors are plotted. **c**) For beta-CoV and SARS-CoV-2 the proportion of clones that are reactive towards S1 (orange) or S2 (dark red) are indicated.

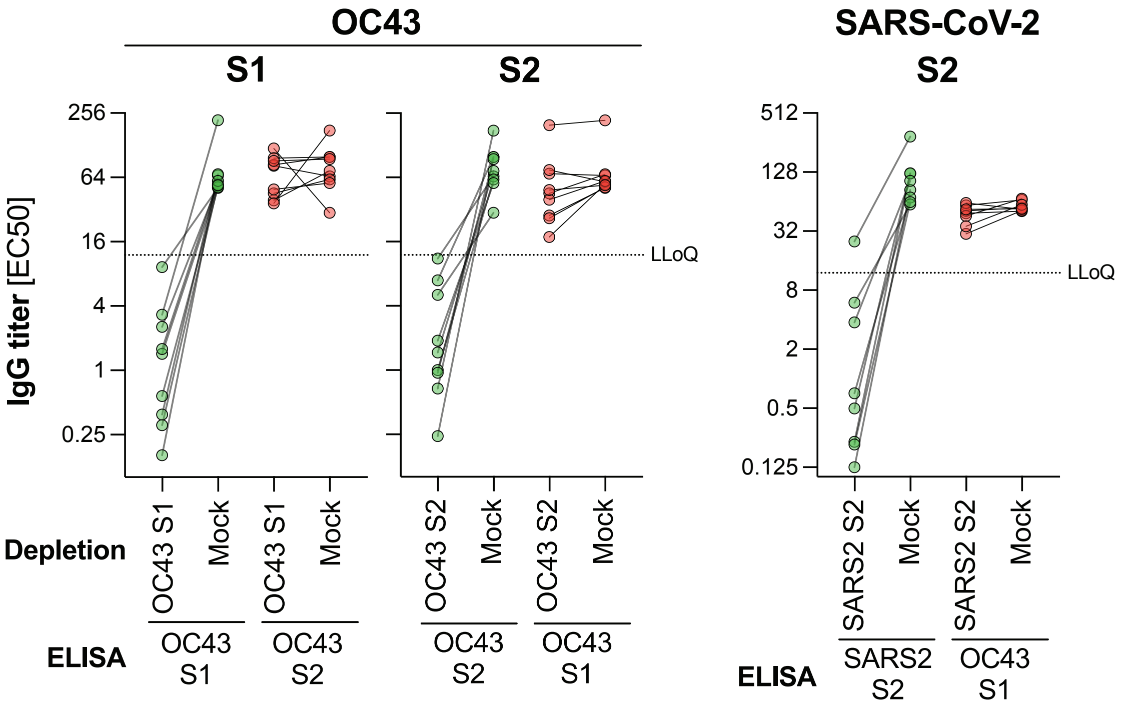


**Supplementary Figure 2. Depletion of OC43 S1, S2 and SARS-CoV-2 S2-specific antibodies from serum.** Antibodies specific for OC43 S1 (left panel), S2 (middle panel) and SARS-CoV-2 S2 (right panel) were depleted from end-pandemic sera using antigen coated magnetic beads. Sera treated analogously with uncoated beads (mock) were used as a comparator. Efficiency and specificity of depletion was confirmed by determining the 50% endpoint titer (EC50) of homologous and heterologous IgG using ELISA, respectively. The dotted line indicated the lower limit of quantification.

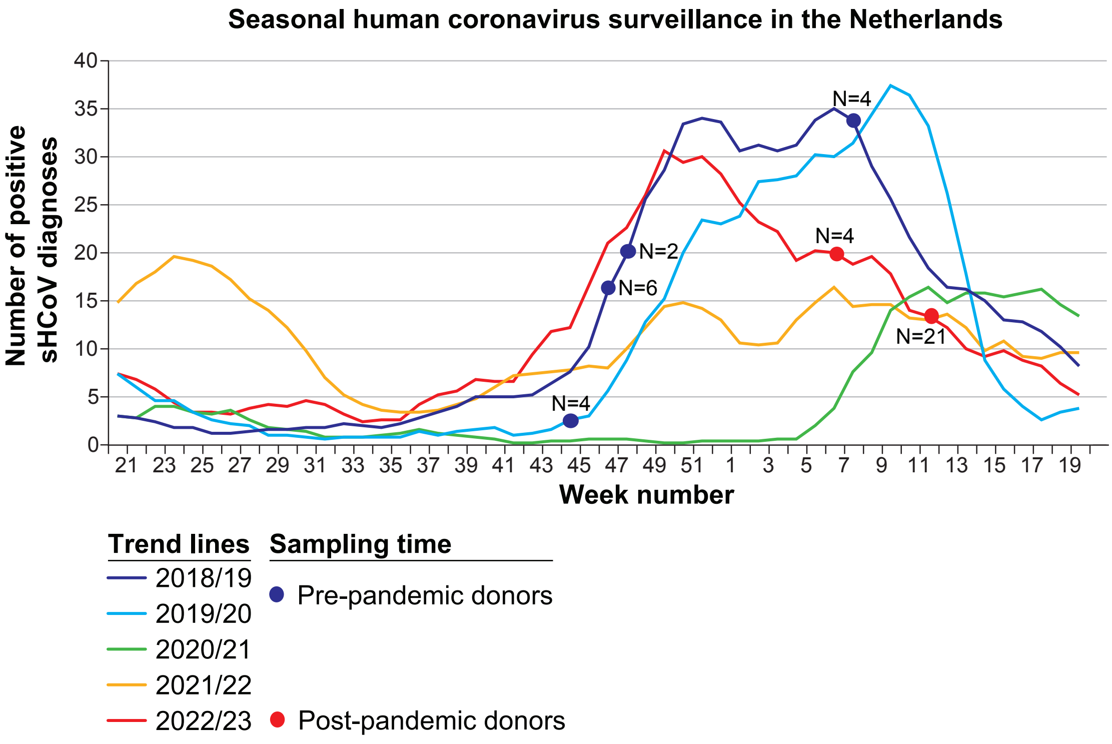


**Supplementary Figure 3. Seasonal coronavirus surveillance in the Netherlands.** 5-week moving average trendlines for the number of reported positive test results for seasonal coronaviruses (excluding SARS-CoV-2) in the virological laboratory surveillance in the Netherlands for the seasons 2018/19 up to 2022/23. Sampling time and number of donors included in our pre- and end-pandemic cohorts are indicated on the respective trendline. [Figure adapted from: *DFM Reukers et al. (2023), Annual report surveillance of acute respiratory infections in the Netherlands; winter 2022/2023. National Institute for Public Health and the Environment*]

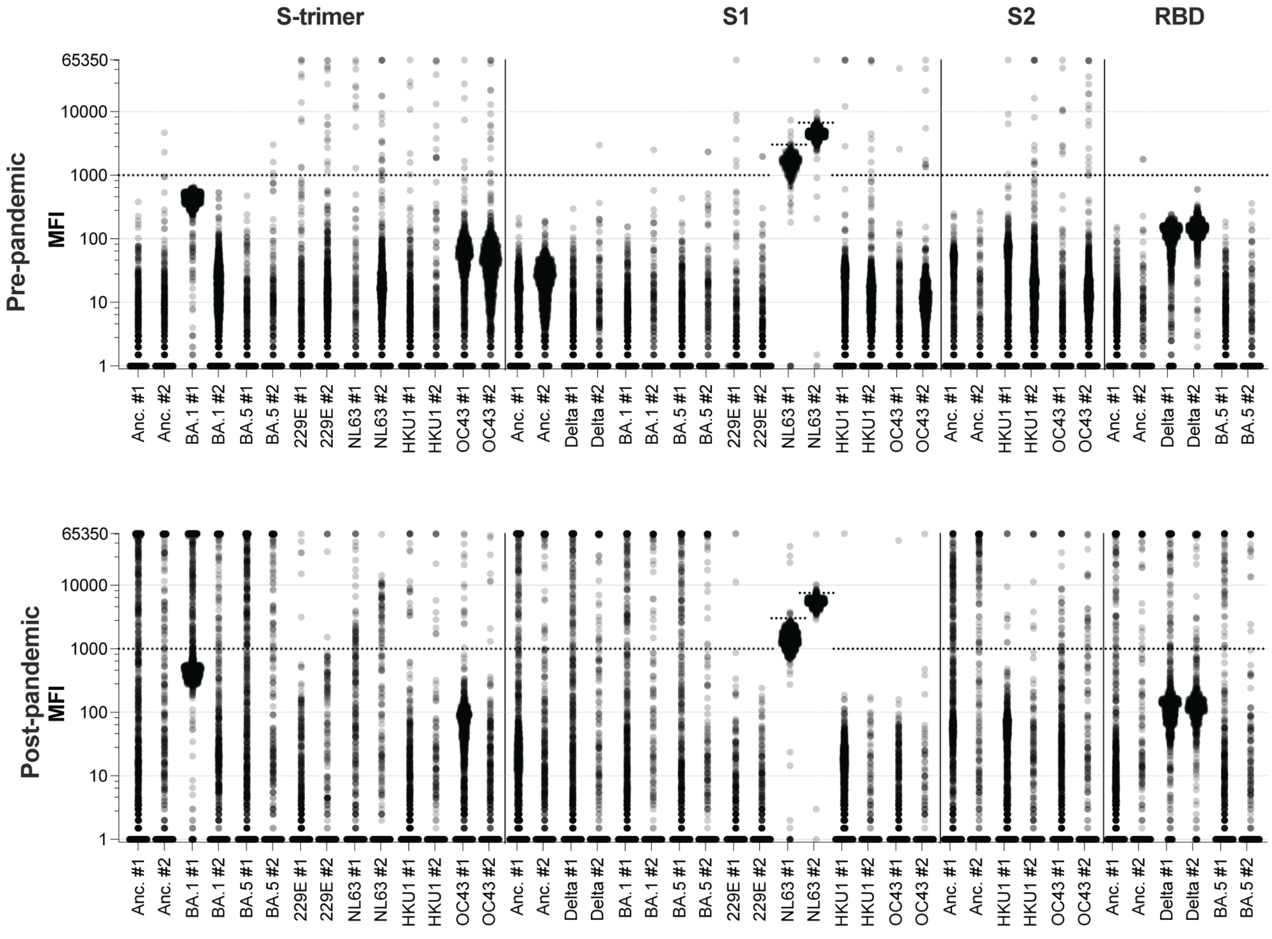


**Supplementary Figure 4. Background corrections for protein microarray per antigen.** The mean fluorescent intensity (MFI) of two antigen spots per antigen were plotted to define fluorescence cut-offs for positive cultures per antigen and per batch of slides (#1 and #2). A cut-off of 1,000 MFI was set for all antigens, except for NL63 S1, due to a higher background signal. A batch-dependent cut-off was set for this antigen at 3,000 MFI for batch #1 and 6,000 MFI for batch #2 based on the MFI of non-reactive supernatants.
